# The Impact of Intraspecies and Interspecies Bacterial Interactions on Disease Outcome

**DOI:** 10.3390/pathogens10020096

**Published:** 2021-01-21

**Authors:** Jiwasmika Baishya, Karishma Bisht, Jeanette N. Rimbey, Kiddist D. Yihunie, Shariful Islam, Hafij Al Mahmud, Jayc E. Waller, Catherine A. Wakeman

**Affiliations:** Department of Biological Sciences, Texas Tech University, Lubbock, TX 79409, USA; jiwasmika.baishya@ttu.edu (J.B.); karishma.bisht@ttu.edu (K.B.); jeanette.rimbey@ttu.edu (J.N.R.); kiddist.yihunie@ttu.edu (K.D.Y.); shariful.islam@ttu.edu (S.I.); hafij-al.mahmud@ttu.edu (H.A.M.); jayc.waller@ttu.edu (J.E.W.)

**Keywords:** polymicrobial infection, host–pathogen interface, pathogen, commensal, bacterial interactions

## Abstract

The human microbiota is an array of microorganisms known to interact with the host and other microbes. These interactions can be competitive, as microbes must adapt to host- and microorganism-related stressors, thus producing toxic molecules, or cooperative, whereby microbes survive by maintaining homeostasis with the host and host-associated microbial communities. As a result, these microbial interactions shape host health and can potentially result in disease. In this review, we discuss these varying interactions across microbial species, their positive and negative effects, the therapeutic potential of these interactions, and their implications on our knowledge of human well-being.

## 1. Introduction

Microbes constitute an integral part of human life and impact human health through intraspecies, interspecies, and interkingdom interactions. These interactions result in a broad spectrum of outcomes for the human host, ranging from beneficial to pathogenic. Diseases were once believed to originate from a single species of pathogenic microorganisms. However, following the advent of next generation sequencing technologies and related techniques, several pathological conditions are now being considered as an outcome of multiple microbial species. Many pathogenic species in such communities exhibit unique strategies to circumvent the host immune responses as well as the potential stressors produced by competing commensal microorganisms in order to establish long-term infections [1]. One way they accomplish this is by producing signaling molecules in their extracellular environment. Molecules of this nature can serve as cues for escaping host immune responses, outcompeting nearby microbes [2], and aiding in subpopulation differentiation to combat multiple stressors and antimicrobial components [3,4]. Another way by which pathogenic species survive in the host environment is by exhibiting cooperative/synergistic behavior, such as sharing resources to reduce overall energy expenditure or exploiting the virulence factors of neighboring pathogens [5].

It is also important to acknowledge that microbial interactions at the host–pathogen interface can occur indirectly and directly [1]. Indirect interactions can occur as a result of environmental changes influenced by the presence of other microbes and can often be mediated by immune responses at the host–pathogen interface [6] and direct interactions can include interactions, such as competition between microorganisms in which they actively produce toxic substances to kill or limit colonization of other microbes [1,7]. The microbes being outcompeted may include beneficial or native microbes of the host microbiota but may sometimes also include invading pathogens. Therefore, microbial interactions at the host–pathogen interface can affect the host positively as well as negatively depending upon the nature and characteristics of the existing interactions [8,9,10]. For example, both beneficial and detrimental microbial interactions can be found in multiple host niches including the oral cavity and the gastrointestinal tract and are especially apparent during chronic illnesses such as cystic fibrosis infections, diabetic foot wounds, and otitis media diseases [11]. Comprehending interactions within the diverse polymicrobial populations existing within humans will improve our predictions regarding disease severity and outcome [11]. In this review, we discuss some of such critical microbial mechanisms and their role in disease progression.

## 2. Intraspecies Interactions Can Elevate Microbial Virulence: Pathogenic Communities Communicate to Coordinate Behavior and Establish Infection

**Figure 1:**
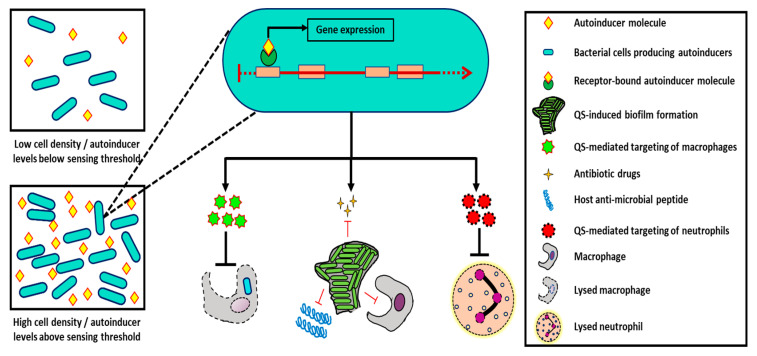
Quorum sensing-mediated intraspecies interactions in bacteria can elevate pathogenesis in host tissues. AHL-bound autoinducers in Gram-negative bacteria like *P. aeruginosa* can promote the expression of several virulence factors such as rhamnolipids, pyocyanin, and other biofilm-associated genes. Some of these factors can target host immune cells such as macrophages upon phagocytosis. Similarly, QS-mediated biofilm-associated genes can prevent the elimination of pathogenic cells from host tissues by resisting antibiotic effects of antimicrobial peptides, antibiotic drugs, and phagocytic immune cells. Additionally, QS-induced virulence factors including proteases produced by Gram-positive bacteria, such as *S. aureus*, can degrade immune system cells upon infection.

Quorum sensing (QS) is a bacteria’s communicating system wherein they utilize small hormone-like molecules, referred to as autoinducers, to coordinate population behavior in response to the changing environment [12]. These communicating systems can regulate genetic expressions and enable intraspecies interactions as discussed in this section [13,14]. QS-mediated intraspecies interactions have been shown to change the course of host infection by regulating virulence factors that impact the ability of pathogens to evade host immune responses (Figure 1).

*Pseudomonas aeruginosa* is one of the human pathogens whose QS-regulated virulence has been particularly well characterized. For example, in one study, in vitro and in vivo experiments conducted with QS-deficient *P. aeruginosa* strains demonstrated that N-acyl homoserine lactones (AHL)-mediated QS is vital in upregulation of *P. aeruginosa*’s virulence factors such as elastase, rhamnolipids, and pyocyanin [15]. The presence of these virulence factors can trigger the host’s toll-like receptor response and, in turn, upregulate expression of *P. aeruginosa*’s genes that are responsible for limiting detection by host inflammatory responses [16]. This effect is exacerbated by the hemolytic activity of the QS-regulated rhamnolipids produced by *P. aeruginosa* that have been shown to lyse macrophages and polymorphonuclear leukocytes in multiple studies [17,18] (Figure 1). Lysis of these types of leukocytes can also be mediated directly by certain *P. aeruginosa* AHLs [19]. Such mechanisms can confer resistance to *P. aeruginosa* from host responses and allow it to colonize host tissues successfully [16]. Much like *P. aeruginosa*, *Vibrio cholerae*, another pathogenic Gram-negative microorganism, is known to utilize its cholera autoinducer-1 (CA-1) QS molecule for coordinating expression of virulence genes, hemagglutinin/protease genes, and genes necessary for biofilm formation [20] (Figure 1). Expression of such genes aids in the progression of *V. cholerae*’s persistent infection in the human host.

In addition to these AHL-based QS molecules, non-AHL, diffusible signaling molecules have also been shown to assist some Gram-negative bacteria during host infections. For example, the diffusible signaling molecule, cis-2-dodecenoic acid, also known as Burkholderia diffusible signal factor (BDSF), is a non-AHL signaling molecule involved in intraspecies communication and virulence regulation in *Burkholderia cenocepacia* [21]. BDSF has been shown to regulate biofilm formation and motility in *B. cenocepacia* and is responsible for exacerbating disease progression in infected hosts [22].

In Gram-positive bacterial species, like *Staphylococcus aureus*, the Agr QS system regulates attachment, colonization, dissemination, toxin secretion, and virulence factor expression of the pathogen [23,24]. Although virulence factors produced by *S. aureus* are recognized by the host receptor TLR2, *S. aureus* can often evade the host due to the expression of multiple host immune-counteracting molecules [25]. For example, QS induced virulence factors, such as proteases, allow *S. aureus* to degrade immune system components [26] (Figure 1). At the same time, other proteins enable the pathogen to inhibit the antimicrobial functions of specific host immune components [26]. Such QS-mediated mechanisms aid *S. aureus* in infection establishment by allowing it to combat host immunity and induce inflammatory responses, both of which are detrimental to human health.

In addition to *S. aureus*, QS-mediated intraspecies interactions have been shown to impact the pathogenesis of other Gram-positive bacterial species. In *Streptococcus agalactiae*, an opportunistic Gram-positive pathogen, disruption of the SHP/RovS intercellular communication system results in a significant decrease in the ability of the pathogen to invade and adhere to human hepatic cells [27]. The SHP/RovS is *S. agalactiae*’s cell-to-cell communication system that consists of a transcriptional regulator from the Rgg family and short hydrophobic peptides (SHPs), which act as signaling molecules. In some Group A *Streptococcus* pathogens, upregulation of this communication system provides resistance to lysosomal killing of pathogens during metal deficient and altered carbon source conditions [28].

In addition to acting as signaling molecules that regulate virulence factors, QS systems can also impact cell growth and lifestyle choices in pathogenic bacteria. These choices can, in turn, influence the progression and nature of infection in the host. For example, *S. aureus* can differentiate into dispersal cells and cause acute bacteremia or form biofilm structures that cause chronic infections. A study showed that differentiation of *S. aureus* is antagonistically regulated by the bimodal switch of the Agr QS system. The Agr bimodal switch is initiated by an elevated concentration of autoinducing peptides (AIPs) [29]. Once activated, this switch triggers two adjacent promoters responsible for cell differentiation [29]. Taken together, these selected examples highlight the crucial role of bacteria’s QS-mediated intraspecies communication in host colonization and infection establishment. Numerous additional QS systems in diverse microbial pathogens exist to facilitate host colonization and evade the host immune responses.

## 3. Intraspecies Interactions Can Benefit Human Health: Commensal Strains Can Outcompete Pathogenic Strains

Multiple strains of the same species can frequently interact with each other at the host–pathogen interface. While the previous section highlighted mechanisms by which intraspecies interactions enable the coordination of community behavior to promote host colonization, intraspecies interactions between divergent strains can often be competitive in nature as these strains compete for resources in the same host niche. In some instances, one or few of these strains could be virulent while others remain avirulent. If the avirulent strain outcompetes the virulent strain, a positive host outcome can be achieved. This section brings into light some intraspecies interactions that can be attributed to impacting the host health positively.

**Figure 2:**
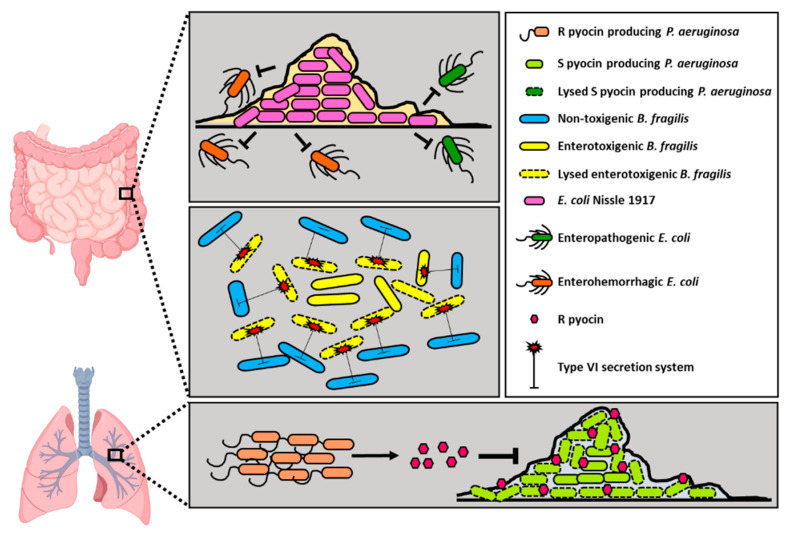
Intraspecies interactions can alleviate pathogenesis in host tissues. *E. coli* Nissle 1917 strain outcompetes enterohaemorrhagic and enteropathogenic *E. coli* by forming highly robust biofilms and reducing the colonizing area for the latter in host tissues (**top panel**). Non-toxigenic *B. fragilis* strains colonizing the gut use their T6SS to lyse enterotoxigenic *B. fragilis* and limit colonization of the pathogenic strain (**middle panel**). In CF lungs, R pyocin producing *P. aeruginosa* strains can inhibit S pyocin producing ones by targeting biofilm communities of the latter (**bottom panel**). Created with BioRender.com.

Multiple biofilm-forming *Escherichia coli* strains are known to be responsible for causing a wide range of gastrointestinal infections within the human gut [30]. One strain of particular interest, Nissle 1917, has been shown in multiple studies as a probiotic that can alleviate intestinal disorders [30]. It was discovered that Nissle 1917 is better at biofilm formation and can outcompete other enteropathogenic, enterotoxigenic, and enterohaemorrhagic *E. coli* strains (Figure 2). Although Nissle 1917 was unable to outcompete the planktonic growth phase, it could outcompete other *E. coli* strains, MG1655, EPEC 1020, and ETEC H10407, during biofilm growth phase [30]. Nissle 1917′s ability to do so has been attributed to its ability to use a mixture of six sugars during nutrient-limiting conditions in the intestine.

Additional examples of such intraspecies competition can be observed in studies involving enterotoxigenic *Bacteroides fragilis* (ETBF), a causative agent of inflammatory bowel disease (IBD) [31]. A study investigated the ability of symbiotic non-toxigenic *B. fragilis* strains in limiting colonization of a murine host by pathogenic ETBF via the type VI secretion system (T6SS) (Figure 2) [31]. This study demonstrated competitive exclusion of ETBF by the non-toxigenic *B. fragilis* strain. It also showed that a non-toxigenic *B. fragilis* mutant lacking a key mechanism for T6SS, N1 Δ*tssC*, was co- colonized with ETBF within a mouse model system. The non-toxigenic *B. fragilis* strain limited ETBF’s toxin exposure to the host and protected it against IBD.

In addition to the gut microbiome, intraspecies competition has been observed in other parts of the human body, such as in the cystic fibrosis (CF) lung. Strain diversity has been shown within *P. aeruginosa* populations of the CF lung by multiple studies [32,33,34]. Many different strains of *P. aeruginosa* produce molecules called pyocins that can potentially exhibit antimicrobial properties capable of killing other *P. aeruginosa* strains. Studies have shown that R type pyocin-producing strains dominate over non-producers in both planktonic and biofilm lifestyles [35] (Figure 2). While it is improbable that pyocin-producing isolates of *P. aeruginosa* will ever be used therapeutically to target pyocin-sensitive isolates, purified pyocins have the potential to be a therapeutic strategy. These highlighted research studies indicate that intraspecies interactions among bacteria can impact host health positively, and some of these interactions may be potentially exploited to be developed as treatment options in clinical settings.

## 4. Interspecies Interactions Can Exacerbate Disease: Polymicrobial Communities Can Synergize during Infection

As next generation sequencing-based studies improve, it has become apparent that many infections are associated with complex microbial communities [5]. The different species within these sites of infection exhibit complex interactions ranging from mutualistic to antagonistic [36]. In past studies, interspecies interactions have focused mainly on growth-inhibitory interactions [37,38,39] but in this section, we concentrate on examples of synergistic interspecies interactions mediated by factors such as shifts in biofilm developmental processes, production of secondary metabolites, etc. as well as explore their impacts on host health [40].

**Figure 3:**
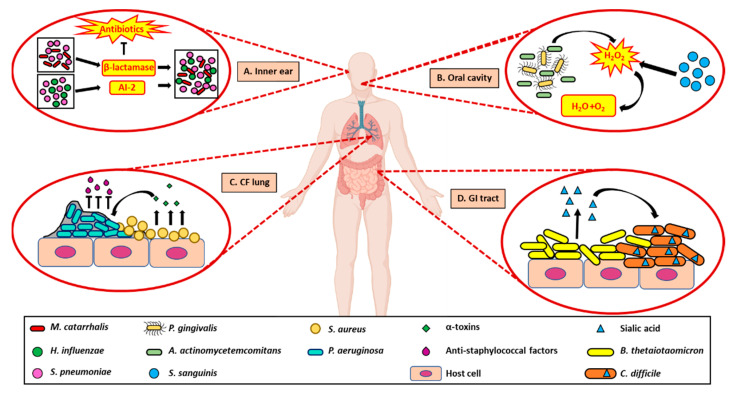
Interspecies interactions can elevate pathogenesis in host tissues. (**A**). In inner ear infections such as otitis media, causative pathogen *M. catarrhalis* produces β-lactamase which protects *S. pneumoniae* from host antibacterial molecules. An increase in the cell density of *S. pneumoniae*, in turn, leads to increased production of auto-inducer AI-2 (via QS) and increases colonization of *M. catarrhalis*. This positive feedback loop contributes to increased antibacterial resistance in pathogenic microorganisms. (**B**). In the oral cavity, *A. actinomycetemcomitans* secrete cytoplasmic catalases that convert H_2_O_2_ produced by *S. sanguinis* to non-lethal end-products and protects pathogenic species, *P. gingivalis*, from the effects of reactive oxygen species. (**C**). In the infected CF lung, reduction in the expression of anti-staphylococcal factors secreted by *P. aeruginosa* and production of molecules such as α-toxins that can counteract the host defenses by *S. aureus* can lead to increased survival of both the pathogenic species. (**D**). In the GI tract, presence of *B. thetaiotaomicron*, a symbiotic bacterial species which produces sialic acids, helps pathogenic *C. difficile* to colonize and grow in the gut by utilizing the acids as a nutrient source. Created with BioRender.com.

Microbial synergism negatively impacting host health has been observed in patients diagnosed with otitis media or inner ear infections. Opportunistic nasopharynx pathogens, *Haemophilus influenzae*, *Streptococcus pneumoniae*, and *Moraxella catarrhalis*, cause otitis media [41] and these microorganisms, via QS, can exacerbate upper respiratory tract infection by decreasing their antibacterial susceptibility [42]. This protection is attributed to the β-lactamase production by *M. catarrhalis*, which protects *S. pneumoniae* from antibacterial agents [43]. Additionally, the increased production of an autoinducer, AI-2, which is involved in QS of *S. pneumoniae*, increases colonization of *M. catarrhalis* significantly during co-infection (Figure 3A). Furthermore, the increase in the colonization of *M. catarrhalis* was shown to slow or delay further ascension of *S. pneumoniae* into the middle ear of the host, which could be considered a potential strategy to prevent immediate clearance from the host [43].

Cooperative interactions negatively impacting host health can also be found in the oral cavity where pathogenic microbial species, *Porphyromonas gingivalis* and *Aggregatibacter actinomycetemcomitans* are known to co-colonize and cause chronic periodontitis. *A. actinomycetemcomitans* possesses a cytoplasmic catalase capable of effectively reducing hydrogen peroxide (H_2_O_2_) produced by other oral microbial species, such as *Streptococcus sanguinis*, resulting in the enhanced survival of *P. gingivalis* [44]. Thus, the presence of *P. gingivalis* and *A. actinomycetemcomitans* together accelerates periodontal disease as the H_2_O_2_-reducing capabilities of *A. actinomycetemcomitans* indirectly allow increased proliferation and protection of *P. gingivalis* (Figure 3B).

Pathogenic synergy has also been witnessed in the lungs of CF patients where co-existence and establishment of chronic lung infection often occurs [5,45]. Under normal circumstances, *P. aeruginosa* and *S. aureus* have an antagonistic relationship wherein *P. aeruginosa* secretes toxins that promote active killing of *S. aureus*. However, in most CF lung infections, overproduction of alginate has been shown to reduce the expression of anti-staphylococcal agents produced by *P. aeruginosa*, aiding in the survival of *S. aureus* [46]. Moreover, the virulence factors secreted by *S. aureus* assist in the proliferation and dissemination of *P. aeruginosa* by counteracting the components of the host immune system [47] (Figure 3C). Such cooperation allows *P. aeruginosa* and *S. aureus* to establish infections that are recalcitrant to known therapeutics [48].

*P. aeruginosa* and *S. aureus* can also act synergistically in host niches outside of the human lung. For example, wounds infected by both of these organisms experience impaired healing due to the combined action of the virulence factors of *P. aeruginosa* and *S. aureus* [10]. Other recent studies highlight cooperative interactions between the wound pathogens *Enterococcus faecalis* and *E. coli* [49]. In this study, it was found that ornithine produced by *E. faecalis* mediated the co-existence of the microbe with *E. coli* in a murine model. Under iron-deficient conditions, ornithine prompts biofilm production in neighboring *E. coli* by favoring metabolic pathways that lead to siderophore synthesis. Increased siderophore synthesis triggers dramatic uptake and iron utilization by *E. coli* resulting in bacterial survival [49]. In general, these studies highlight that synergistic interactions in wound infections can accelerate injury severity and/or facilitate polymicrobial growth.

Unsurprisingly, the complex microbial make-up of the human intestinal tract is also subject to pathogenic synergy [50]. An example of this type of interaction occurs between *Clostridium difficile*, an opportunistic enteric pathogen, and *Bacteroides thetaiotaomicron*. In the presence of the symbiont *B. thetaiotaomicron*, *C. difficile* can expand and accelerate growth in the gut throughout an infection. *B. thetaiotaomicron* is found in the gut mucosa and produces sialic acids that *C. difficile* can metabolize and utilize as a nutrient source (Figure 3D). This interaction enables *C. difficile* to colonize this niche rapidly [51]. These are only a small subset of the complex pathogenic polymicrobial interactions that have been uncovered in recent years, demonstrating how the synergistic interaction between microbes can have an adverse effect on human host and worsen disease progression. However, unlike the above-mentioned detrimental interspecies interaction, there are also some known interspecies interactions that can be beneficial to the human host and can benefit human health.

## 5. Interspecies Interactions Can Benefit Human Health: Commensal Species Can Outcompete Pathogenic Species

**Figure 4:**
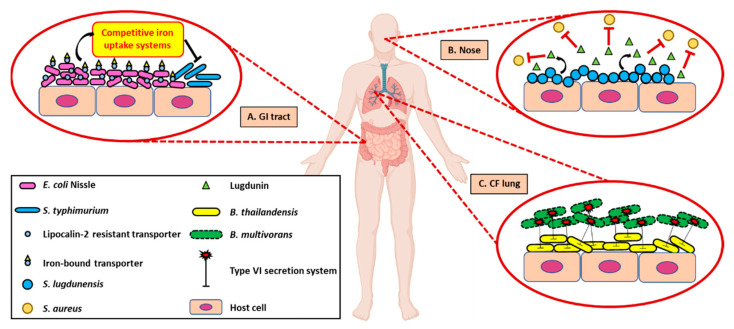
Interspecies interactions can alleviate pathogenesis in host tissues. (**A**). Commensal microorganisms, including *E. coli* Nissle 1917, can outcompete growth of pathogenic bacteria such as *S. typhimurium* via competitive iron acquisition in the human gut. Such mechanisms exhibited by non-pathogenic microbes help in alleviating pathogenesis of the human host. (**B**). Antibiotic peptide, lugdunin, produced by *S. lugdunensis*, a resident bacterial species, inhibits colonization of *S. aureus* in the human nasal cavity. (**C**). In CF lung, non-pathogenic *B. thailandensis* cells target *B. multivorans* by delivering toxins via T6SS contact-dependent growth inhibition, thereby killing the pathogenic species. Created with BioRender.com.

While microbiology studies often focus on the pathogenic microbes that are detrimental to human health, the impact of the human microbiota is typically positive for the well-being of the host. One of the major ways in which the microbiome can benefit its host is through suppression of competing microbes that would otherwise act as pathogenic invaders. *Salmonella typhimurium*, one of the causative agents of acute gastritis, is exceptionally well adapted to acquire iron in an inflamed gut [52]. It can thrive within this environment by changing the siderophore it uses to evade the host produced lipocalin-2 used to sequester many microbial siderophores [52]. *E. coli* strain Nissle 1917, discussed earlier as exhibiting intraspecies competition that is beneficial to the host, has been shown to outcompete and limit intestinal colonization of *S. typhimurium* by secreting similar siderophores that are resistant to lipocalin-2 (Figure 4A) [52].

Commensal bacteria are known to be found at various sites in the human body. One such site where it plays a major role in keeping away infection is the nasal cavity. For example, *Staphylococcus lugdunensis*, a resident bacterial species colonizing the human nose along with *S. aureus*, can produce the antibiotic peptide lugdunin, to prevent *S. aureus* from colonizing the human nose. The bactericidal activity of lugdunin acts against clinical isolates of *S. aureus* as well, demonstrated by inhibition of *S. aureus* colonization in a mouse model of skin infection (Figure 4B) [53]. Another recent example highlighted the role of *S. epidermidis* in promoting a healthy microbiome by producing antimicrobial peptides, resulting in a low pathogen appearance in the nasal cavity [54]. Additionally, *Neisseria lactamica*, another commensal bacterial species, within the nasal cavity, is known to exhibit protective properties against infection by *Neisseria meningitidis*, the causative agent of meningitis, by reducing its ability to colonize the nasal cavity, possibly through triggering the host innate immune responses [55].

Similarly, another site where we happen to see competing interactions between the commensal and pathogenic microbes is the lung. Among the known microbial species inhabiting the lung, *Burkholderia multivorans* is a pathogenic one causing pulmonary infections in CF patients and a recent study demonstrated that non-pathogenic *Burkholderia thailandensis* cells could target *B. multivorans* by delivering toxins via contact-dependent growth inhibition, thereby killing the pathogenic species (Figure 4C) [56]. Additionally, the discovery of lung-based probiotics is also of interest to the community since probiotic consumption can influence the overall lung microbiota, decreasing the chance of respiratory tract infections [57]. Understanding the protective mechanisms employed by commensal microorganisms can aid in the creation of future probiotics tailored to treat a specific disease.

The human gut is home to several hundred bacterial species, including both commensal and pathogenic microbes. The intestinal microenvironment can create favorable conditions for certain commensal microbes; however, the opportunistic pathogens are known to take advantage of it, eventually leading to a gut microbiota dysbiosis [58]. In order to treat and prevent multidrug-resistant infections in the gut, commensal strains of *E. faecalis* produce bacteriocin. Bacteriocin produced by this commensal strain was able to influence niche competition in the mouse gut reducing the enterococci infection rate [59]. In another study, *S. typhimurium* induced colitis was reduced in the mice gut in the presence of *Mucispirillum schaedleri*, which was known to hamper its virulence factor expression. *M. schaedleri* is a member of the phylum Deferribacteres and is present in the intestinal microbiota of both mice and humans [60]. Overall, these studies highlight the importance of commensal microorganisms and commensal-derived competitive factors as potential treatment strategies that can be used in the future for the eradication of pathogenic bacteria.

In addition to this, interspecies interactions between commensal microbial species and pathogenic microbial species can also induce innate immunity defenses against the latter. For example, intestinal commensal bacterial species have been shown to contribute to the host adaptive immunity via generation of T-cell subsets. Many studies have supported such effects on the immune responses by the host’s commensal bacteria [61,62]. Thus, beneficial microorganisms can reduce pathogenic colonization by either aiding the host’s innate/adaptive immune responses or by directly inhibiting pathogen colonization [63].

## 6. Conclusions

Neighboring microorganisms interact with each other and the host environment. These microbe–microbe and microbe–host interactions can determine the characteristics of the microbial community and the health of the host. Several recent studies have confirmed that many bacterial species inhabiting the human body have evolved to form diverse intra/interspecies microbial communities [1,64]. Colonization of diverse bacterial populations within humans can occur in healthy and diseased states leading to beneficial and detrimental microbial interactions within the host [1,11,65,66]. These interactions can impact the host’s immune responses and the overall health of the host. Hence, it is imperative to understand the impact of bacterial communities and their interactions on human health and diseases.
